# Current Public Trends in the Discussion of Dry Eyes: A Cross-Sectional Analysis of Popular Content on TikTok

**DOI:** 10.7759/cureus.22702

**Published:** 2022-02-28

**Authors:** Shahrukh Naseer, Sazid Hasan, Julia Bhuiyan, Anuradha Prasad

**Affiliations:** 1 Ophthalmology, Oakland University William Beaumont School of Medicine, Rochester Hills, USA; 2 Ophthalmology, Harvard University, Cambridge, USA; 3 Ophthalmology, Beaumont Hospital, Royal Oak, USA

**Keywords:** discern, cross-sectional analysis, tiktok, social media, dry eyes

## Abstract

Introduction

Dry eye disease (DED) is a common ocular pathology with significant impacts on both quality of life and visual function. One platform where individuals are receiving healthcare information is TikTok, the world's fastest-growing social media platform. Though used by more than one billion users, current literature is not established to assess the quality of information on TikTok. The purpose of this study was to assess the quality of DED-related medical information present on TikTok.

Methods

We conducted a cross-sectional analysis of DED content on TikTok, utilizing the search term #DryEye to assess the top 150 videos appearing on December 20, 2021. Included videos were analyzed for descriptive statistics, including views, likes, uploader profession, and the number of uploader followers. Videos were assessed utilizing DISCERN, a tool used to appraise consumer health information. The one-way analysis of variance (ANOVA) was used to determine statistical significance groups.

Results

A total of 101 videos were included in the final analysis. When comparing content creators, physicians received a significantly greater number of views and higher DISCERN scores (p<0.05) than non-physician medical providers and non-medical individuals. The content of the videos were educational content (n=39, 38.6%) or treatment information (n=37, 36.6%), followed by home remedies (n=10, 9.9%) and personal anecdotes (n=8, 7.9%). Videos with rich supplementary visuals (multiple images/moving images) had higher DISCERN scores compared to videos with no supplementary visuals or one supplementary visual (p<0.01).

Conclusion

With the growing popularity of TikTok, it is important to provide high-quality information to ensure the dissemination of medically accurate information and reduce the prevalence of disinformation. Our results demonstrate that while TikTok is a powerful platform, the quality of videos can still be vastly improved. Content creators, regardless of profession, can improve their DISCERN through listing sources, comparing treatments, and discussing risks/outcomes of various treatment modalities.

## Introduction

Dry eye disease (DED) is a commonly encountered chronic pathology in ophthalmic practice characterized by a reduction in the quality of the tear film [1]. With an estimated prevalence of between 5% and 50% of the adult population, DED is an ever-growing public health concern with a significant socio-economic impact [1-2]. It has been shown to tremendously affect the quality of life, as symptoms can interfere with common activities such as reading, driving, or using electronic screens [3]. At present, depending on the severity and characteristics of the symptoms, there are many different treatment options for DED at a physician's disposal, ranging from over-the-counter treatment options, such as artificial tears, repetitive hot compress, increasing blink repetition, and omega-3 fatty acids to in-office procedures and prescription medications [3-4].

Despite the many treatment options, many individuals affected with DED may not seek professional help for their condition. Within the last decade, there has been an increase in the use of social media platforms amongst physicians and non-physicians alike to disseminate medical knowledge pertaining to specific diseases [5]. One study that randomly surveyed 160 ophthalmologists primarily from the United States demonstrated that upwards of 40% of these physicians use social media for professional reasons, including publicizing their practice, demonstrating expertise in the field, and educating patients and health professionals [6]. Physicians and non-physicians are increasingly using TikTok, an application where users can upload short mobile videos, to impart medical advice [7]. Given that this social media platform already has over one billion active users worldwide, TikTok’s popularity may provide significant opportunities for health communication [8-9].

While TikTok holds tremendous potential as a feasible tool of patient education, the quality of the shared information is a concern [10]. One study found that the majority of videos that they had assessed did not contain high-quality educational content [11-12]. Encountering low-quality medical information on social media platforms may pose dangerous risks for patients, as they may make crucial medical decisions based on potentially faulty information that could have adverse effects. Beyond the aforementioned study, however, current literature on the information quality of these videos is limited and consists primarily of short reports without extensive analysis. Furthermore, there is a lack of investigation into the communication and visual quality of these videos [10].

The quality of information on DED available to the public on social media is not well-characterized. In this cross-sectional study, we seek to contribute to the existing literature by assessing the quality of medical information provided in videos shared on TikTok related to DED with regards to the type of content creator, quality of communication, and video content. With this information, we hope that medical providers, particularly ophthalmologists and optometrists, and the community of content creators at large may be in a more informed position to develop higher-quality content for their amassing viewership of individuals affected by DED.

## Materials and methods

**Figure 1:**
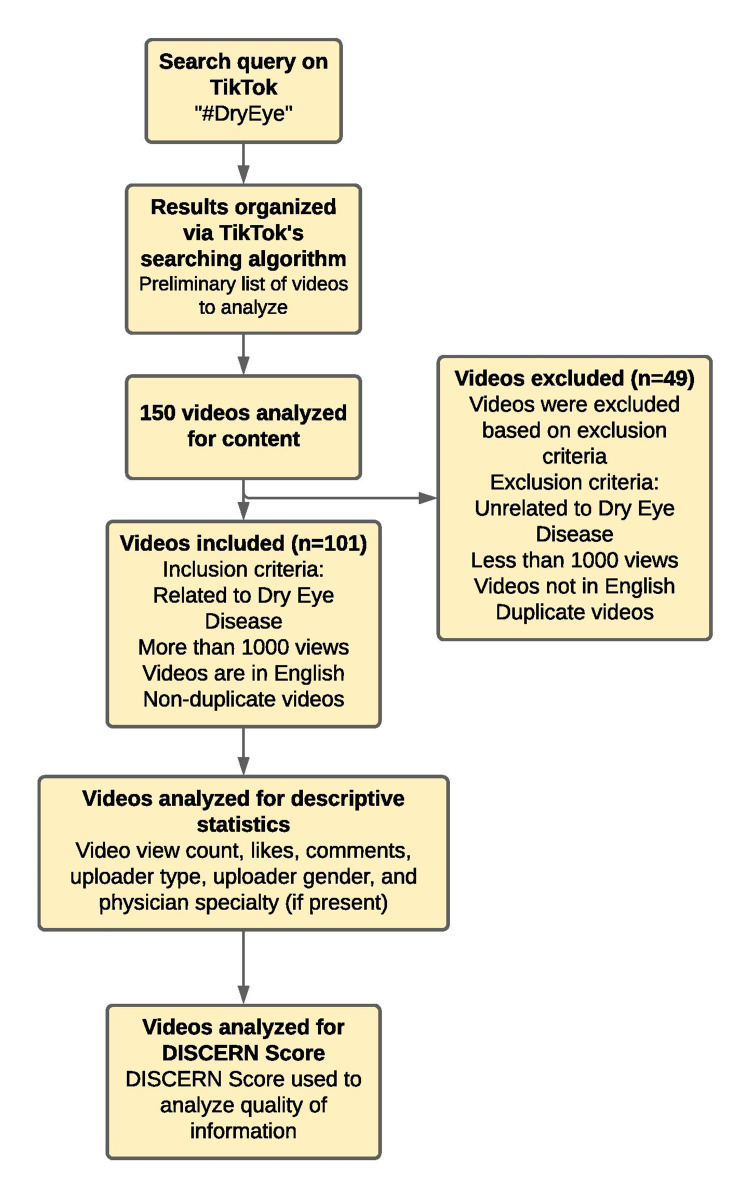
Video Inclusion/Exclusion Flow Diagram

A cross-sectional analysis of DED content on TikTok was conducted on December 20, 2021, using the #DryEye search term. The 150 most popular videos related to DED were chosen based on our inclusion criteria and included in our analysis. Videos were included if they discussed or presented anything relevant to DED, had a minimum of 1,000 views, and were in English (Figure 1). Forty-nine videos were excluded, as they were unrelated to DED, had less than 1,000 views, were not in English, or were duplicates. For each video, descriptive statistics were recorded, including video view count, likes, comments, uploader type, uploader gender, and physician specialty if present. Content creators were further categorized into influencer group types based on followers (nano-influencers having one to 10,000 followers, micro-influencers having 10,000 to 50,000 followers, mid-tier influencers having 50,000 to 500,000 followers, macro-influencers having 500,000 to one million followers, and mega influencers having one million to five million followers) [13]. The videos were also categorized on video length: 0-15 seconds, 15-30 seconds, 30-45 seconds, and >45 seconds [13-14]. Videos were then also stratified by the quality of communication and were placed in group 1, group 2, or group 3: group 1 videos had no supplementary visuals, group 2 videos had minimal supplementary visuals (i.e., one image), and group 3 with videos rich in supplementary visuals (i.e., moving image, multiple images). The videos were finally assessed utilizing DISCERN, a tool used to appraise consumer health information on a scale of 1 (poor) through 5 (excellent) for 15 questions. Pearson’s correlation was calculated to determine inter-rater reliability.

Numerical data were summarized as mean ± standard deviation and categorical variables were summarized as n (%). Differences between the medical provider types were examined using an unpaired, independent two-sample t-test for continuous outcomes with normal distributions. One-way analysis of variance (ANOVA) is used to determine statistical significance between the means of three or more independent groups. Tukey's Honest significant difference test was used to calculate differences in mean DISCERN scores between content creators. Statistical significance was set at p < 0.05. Analyses were performed using Microsoft Excel v. 2103 (Microsoft Corporation, Redmond, Washington).

## Results

A total of 101 videos meeting the inclusion criteria were included in our final analysis. The videos had a total of 98,170,746 views, amassing 6,277,452 likes and 65,738 comments. Quality scores utilizing DISCERN ranged from 21 to 50, with a mean DISCERN score of 33.91 with high inter-rater reliability (Cohen's Kappa > 0.9).

When assessing gender distribution, most content creators were female (n=81, 80.2%) while a smaller number were male (n=17, 16.8%) (Table 1). Content creators were most commonly non-physician medical providers (n=65, 64.4%), followed by non-medical individuals (n=17, 16.8%), licensed physicians (n=16, 15.8%), and private companies (n=3, 3.0%). Among these groups, physicians received a significantly greater number of views (p<0.001). Furthermore, DISCERN scores were significantly higher for physicians as compared to other content creators (p=0.0151). Of videos that identified provider type, optometrists had more video content by volume (n=61, 60.4%) than ophthalmologists (n=16, 15.8%) (Table 1). Content creators specializing in ophthalmology received significantly more views (p<0.001), as well as significantly higher DISCERN scores (p=0.0319). There was no significant difference in the number of likes or comments between these provider types.

**Table 1:** Overview of DED Content on TikTok *One-way analysis of variance (ANOVA); ^Two-tailed T-test; DED: dry eye disease

	Total Number of Videos, (%)	Mean Number of Views, (SD)	P-Value	Mean Number of Likes, (SD)	P-Value	Mean Number of Comments, (SD)	P-Value	Mean DISCERN Scores, (SD)	P-Value
Content Creator									
Physician	16 (15.84)	4,784,567 (1,114,133)	P<0.001*	34,930 (87,372)	P=0.864*	257 (421)	P=0.910*	38.25 (6.88)	P<0.01*
Non-physician medical provider	65 (64.36)	1,007,088 (2,777,836)		79,791 (347,239)		835 (4,309)		33.85 (6.69)	
Non-medical Individual	17 (16.83)	1,132,564 (3,655,497)		23,537 (48,262)		311 (693)		30.82 (7.88)	
Private company	3 (2.97)	1,933,711 (2,182,004)		44,010 (56,256)		677 (1,113)		29.67 (2.31)	
Provider Type									
Ophthalmology	16 (15.84)	4,784,567 (1,114,133)	P=0.0001^	34,930 (87,372)	P=0.59^	257 (421)	P=0.58^	38.25 (6.88)	P=0.032^
Optometry	61 (60.4)	1,055,317 (2,861,215)		83,760 (358,188)		882 (4,447)		34.05 (6.83)	
Gender									
Female	81 (80.2)	796,293 (2,509,603)	P=0.425*	63,660 (312,015)	P=0.991*	649 (3,856)	P=1*	33.91 (7.03)	P=0.54*
Male	17 (16.83)	1,639,405 (3,680,726)		58,173 (92,629)		656 (1,009)		34.65 (8.17)	
Private company	3 (2.97)	1,933,711 (2,182,004)		44,010 (56,256)		677 (1,113)		29.67 (2.31)	
Video Types									
Home Remedy	10 (9.9)	102,586 (99,071)	P=0.548*	5,001 (5,494)	P=0.689*	82 (88)	P=0.336*	32.80 (7.44)	P=0.0014*
Educational Content	39 (38.61)	1,223,030 (3,241,257)		115,385 (441,418)		1,254 (5,540)		33.31 (4.74)	
Personal Anecdote	8 (7.92)	722,227 (965,961)		32,212 (43,350)		2856 (452)		25.63 (9.15)	
Product Advertisement	7 (6.93)	2,272,758 (5,659,796)		34,862 (63,176)		552 (1,011)		33.57 (7.07)	
Treatment Info	37 (36.63)	806,658 (1,902,198)		35,070 (95,842)		282 (547)		36.74 (7.63)	

The videos were most commonly educational content (n=39, 38.6%) or treatment information (n=37, 36.6%), followed by home remedies (n=10, 9.9%), personal anecdotes (n=8, 7.9%), and product advertisements (n=7, 6.9%) (Table 1). There was no significant difference in the number of views, likes, or comments across these various types of videos. However, DISCERN scores were significantly higher for videos regarding treatment information compared to other video types (p=0.0014).

While there was no significant difference in DISCERN score between most groups, DISCERN scores were significantly lower for non-medical individuals compared to physicians (p=0.0131). There was no significant difference in the number of likes or comments among these groups. With regards to the type of influencer, micro-influencers had the highest mean DISCERN score while macro-influencers had the lowest mean DISCERN score; however, there was no significant correlation between influencer type and DISCERN score (p>0.05).

**Figure 2:**
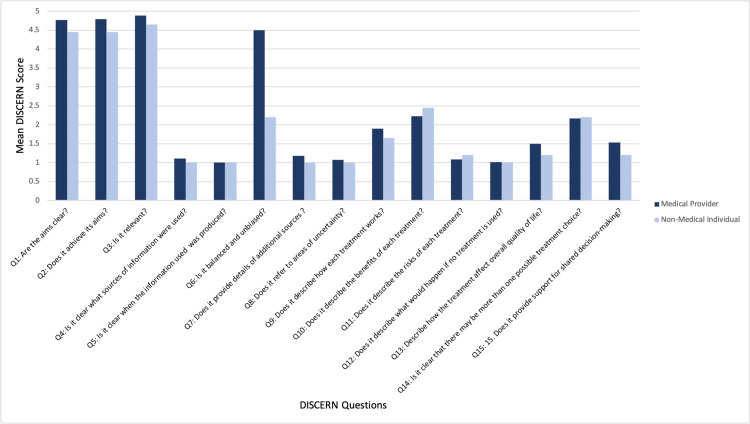
Comparison of Mean Discern Scores Between Medical Providers and Non-Medical Content Creators

Content creators scored highest on DISCERN questions 1 (“Are the aims clear?”), 2 (“Does it achieve its aims?”), 3 (“Is it relevant?”), and 6 (“Is it balanced and unbiased?”) (Figure 2). However, scores were distinctively lower on DISCERN questions 4 (“Is it clear what sources of information were used?”), 5 (“Is it clear when the information used was produced?”), 7 (“Does it provide details of additional sources?”), 8 (“Does it refer to areas of uncertainty?”), 11 (“Does it describe the risks of each treatment?”), and 12 (“Does it describe what would happen if no treatment is used?”) (Figure 2). Medical providers appeared to score higher on most DISCERN questions when compared to non-medical individuals.

With regards to video content, there was no significant correlation between the quality of communication and the average number of views. However, DISCERN scores were significantly higher for videos that were categorized as Category 3 visual quality (rich supplementary visuals, including multiple images or moving images) compared to videos categorized as Category 1 visual quality (no supplementary visuals) or Category 2 visual quality (one supplementary visual) (p=0.0104) (Table 2). In addition, DISCERN scores were significantly higher for videos that were 30 to 45 seconds in duration (p=0.0201) (Table 3). Shorter videos of up to 15 seconds in duration had the lowest mean DISCERN score. 

**Table 2:** Comparison of Quality of Communication and Mean DISCERN Scores *One-way analysis of variance (ANOVA)

Quality of Communication	N	Mean	SD	P
Category 1 (No supplementary visuals)	39	31.87	6.538	P=0.01*
Category 2 (Minimal supplementary visuals)	5	28.8	4.494	
Category 3 (Rich in supplementary visuals)	57	35.52	7.043	

**Table 3:** Comparison of Video Length and Mean DISCERN Scores *One-way analysis of variance (ANOVA)

Video Length (seconds)	N	Mean DISCERN	SD	P
0-15	40	31.075	6.858	P <0.01*
15-30	21	34.762	7.368	
30-45	23	36.261	7.405	
>45	17	35.563	6.460	

Of the videos with treatment information, the most commonly recommended treatment was lifestyle changes (n=31, 37.8%), which included preventative measures (e.g., avoidance of eyeliner and false eyelashes, limited screen time), diet modifications (e.g., omega-3 fatty acid supplements), and daily practices (e.g., increase in blink rate, use of blue-light glasses, 20-20-20 rule in which an individual looks at something 20 feet away for 20 seconds every 20 minutes, and use of eye mask cover while sleeping). This was followed by at-home treatments such as rice socks and warm compresses (n=17, 20.7%). Other recommended treatments included intense pulsed light (IPL) therapy (n=15, 18.3%) and eye drops (n=11, 13.4%). The least recommended intervention was acupressure (n=8, 9.8%).

## Discussion

The use of social media as a source of information has been gaining popularity, especially as individuals are gravitating toward simple, convenient answers rather than attaining a perceived, complex professional recommendation [15]. Applications such as TikTok provide an accessible way for patients to seek medical advice, especially due to its extensive popularity, widespread use among health professionals, and lack of cost. While TikTok has been widely investigated as a channel of health communication for a variety of conditions, the quality of information on DED available to the public through this social media platform is not yet well-characterized. Our results demonstrate that TikTok is a powerful tool for patient education regarding DED. The 101 videos analyzed in our study amassed nearly 100 million views and over six million likes, as well as tens of thousands of comments and shares, evident to TikTok’s tremendous potential for health outreach. Thus, we suggest that it is in the physician's best interest to provide educational social media content for multiple reasons. By providing basic information such as the use of artificial tears as an initial treatment, physicians can help reduce unnecessary office visits. Additionally, most physicians have the ability to provide unbiased content, as opposed to pharmaceutical companies and physicians serving in consulting roles to them.

We identified four main types of content creators, including licensed physicians, non-physician medical providers, non-medical individuals, and private companies. Non-physician medical providers contributed the most videos while private companies contributed the fewest. We found that physicians received a significantly greater number of views and higher DISCERN scores compared to other content creators. This finding is consistent with that of previous studies, which suggest that physicians are more trusted as health and medical information sources among patients as compared to non-physician sources [8,14]. A possible explanation for this is that physicians are objectively more qualified with greater knowledge and understanding of DED due to the extensive preparation required to earn a medical license (i.e., graduating medical school, receiving postgraduate training through residency, passing a rigorous medical licensing examination) [16]. DISCERN scores were significantly lower for non-medical individuals compared to physicians, reflecting the potentially greater quality of information associated with a more extensive comprehension of DED. Our findings that micro-influencers had the highest mean DISCERN score while macro-influencers had the lowest mean DISCERN score, maybe a reflection of popular creators’ abilities to disseminate information of lower quality.

With regards to specific DISCERN categories, all content creators scored distinctively lower on DISCERN questions related to sources of the information they provided, as well as questions concerning treatment risk and necessity. Our findings support those of the existing literature, as social media resources have been found to frequently lack source citation and present opinions as fact [17-18]. To improve on this particular aspect of information quality, content creators can cite relevant sources within the video (e.g., through the TikTok text feature) and address the risks of the proposed treatment. This is further supported by our finding that longer videos of 30 to 45 seconds in duration had significantly higher DISCERN scores; taking the time to address the sources and limitations of the provided information can increase the quality of medical information in videos. It is also important to recognize that despite the importance of lifestyle modifications, the most commonly recommended treatment in videos included in our analysis, this is not in line with step therapy (the practice of beginning treatment of a medical condition with the most preferred method) and standard of care for the treatment of dry eyes.

In response to the lack of exploration of the effects of the video quality of communication, our study investigated the relationship between quality of communication and quality of DED-related health information [8,10]. We found rich visual quality (Category 3) videos that included multiple and/or moving images to be associated with significantly higher DISCERN scores when compared to other videos of less rich visual quality (Category 1 or Category 2). This is supported by the existing literature, which has found the richness of communication to be important in the reception of knowledge on social media, especially as visual communication is practiced with regards to specific settings (i.e., professional health advisory) and audiences, allowing for the contextualization of shared information [19]. Evidently, greater quality of communication can foster increased quality of shared health information, and we encourage content creators to utilize rich supplementary visuals, such as multiple or moving images, to support the information they present.

While our findings provide an introduction to the quality of content uploaded to TikTok, this study has several limitations. Due to the cross-sectional nature of our study, we are unable to establish any causal relationships. Furthermore, our study was limited to videos in English; therefore, our findings cannot be extended to other popular languages in TikTok videos such as Spanish or Chinese. We encourage further investigations with the aim of our study in other languages for a more substantial understanding of the quality of DED-related information shared on TikTok. There is also a lack of demographic information on the videos’ viewers, which would help obtain deeper insight into the extent of health outreach. Lastly, there are several other methods that could be used to appraise consumer health information, including the DISCERN, Quality Evaluation Scoring Tool, and JAMA benchmarks. While our study only used DISCERN in particular due to its successful use in the evaluation of health information shared online, we encourage future studies of this variety in health information to use more evaluation tools to triangulate the validity of our findings.

## Conclusions

Social media has the potential to be a powerful tool for medical education, and we recommend that physicians and healthcare practitioners provide high-quality information to advance healthcare literacy. Physicians can improve the quality of information shared in their videos through various methods, including the use of rich supplementary material, citation of relevant sources, and thorough evaluation of the risks and necessity of proposed treatments. As the quality of information is higher for videos created by licensed physicians, we implore viewers to conduct their own research utilizing evidence-based sources and consult with physicians regarding the treatment for DED.
